# Reduced heterotrophy in the stony coral *Galaxea fascicularis* after life-long exposure to elevated carbon dioxide

**DOI:** 10.1038/srep27019

**Published:** 2016-06-03

**Authors:** Joy N. Smith, Julia Strahl, Sam H. C. Noonan, Gertraud M. Schmidt, Claudio Richter, Katharina E. Fabricius

**Affiliations:** 1 Australian Institute of Marine Science, Townsville, Queensland, Australia; 2 Alfred Wegener Institute Helmholtz Centre for Polar and Marine Research, Bremerhaven, Germany; 3 University of Bremen, Germany; 4 The Carl von Ossietzky University of Oldenburg, Germany

## Abstract

Ocean acidification imposes many physiological, energetic, structural and ecological challenges to stony corals. While some corals may increase autotrophy under ocean acidification, another potential mechanism to alleviate some of the adverse effects on their physiology is to increase heterotrophy. We compared the feeding rates of *Galaxea fascicularis* colonies that have lived their entire lives under ocean acidification conditions at natural carbon dioxide (CO_2_) seeps with colonies living under present-day CO_2_ conditions. When provided with the same quantity and composition of zooplankton as food, corals acclimatized to high CO_2_ showed 2.8 to 4.8 times depressed rates of zooplankton feeding. Results were consistent over four experiments, from two expeditions and both in field and chamber measurements. Unless replenished by other sources, reduced zooplankton uptake in *G. fascicularis* acclimatized to ocean acidification is likely to entail a shortage of vital nutrients, potentially jeopardizing their health and survival in future oceans.

Corals evolved in oligotrophic waters to be mixotrophs, i.e. both auto- and heterotrophs. Autotrophy is the more studied component of the two nutritional modes. However, heterotrophy is just as important, even though its role in coral health is often ignored or underestimated^1^. In addition to supplementing the organic carbon supplied by endosymbiotic zooxanthellae living within their tissue^2^, heterotrophy provides corals with essential micro- and macronutrients that are not attained through autotrophy^3^. These nutrients, including nitrogen and phosphorus, are needed for tissue growth, zooxanthellae regulation, and reproduction^4^,^5^,^6^,^7^,^8^. Corals obtain these nutrients by the uptake of dissolved organic matter^9^, detrital particulates suspended in the water column^10^, bacteria^11^, and zooplankton^12^. Some species of corals increase their reliance on heterotrophy when under stress due to high turbidity^10^,^13^, increased seawater temperatures that lead to the loss of their endosymbionts (coral bleaching)^1^,^14^, and short-term exposure to elevated carbon dioxide (CO_2_) concentrations^15^,^16^. As environmental stressors from anthropogenic causes continue to increase, heterotrophy may become more relevant in the future to maintain coral health. Here, we explore coral heterotrophy with respect to one of the biggest environmental threats of all, ocean acidification. The term ‘ocean acidification’ describes the shift in seawater carbonate chemistry as anthropogenic CO_2_ is absorbed by the oceans^17^. Under ocean acidification, seawater pH and calcium carbonate saturation states are both reduced. The reduced concentration of carbonate ions increases energy demands to maintain rates of calcification and growth, and triggers other physiological and energetic changes^18^.

The number of studies is limited, but some suggest that coral heterotrophy may reduce the impacts caused by ocean acidification^15^,^16^,^19^. Several laboratory experiments show that adult and juvenile corals can maintain calcification rates with heterotrophy under ocean acidification^15^,^19^,^20^. Other studies found that feeding or nutrient loading did not offset the impacts to coral calcification by increased CO_2_^21^,^22^. For example, calcification rates of *Porites rus* reduced during short-term high CO_2_ exposure but were unaffected by the provision of food^23^. It also remains unresolved whether coral heterotrophy may be affected by ocean acidification, and any underlying mechanisms explaining those changes. Previous studies that have investigated the effects of elevated CO_2_ on coral heterotrophy have shown mixed results. For example, *Porites lutea* expanded its polyps more in high CO_2_ waters, perhaps in an attempt to feed more and ameliorate the negative effects of ocean acidification^24^. Also, the corals *Acropora cervicornis* and *Porites rus* displayed increased rates of heterotrophy under elevated CO_2_^15^,^16^, mitigating the adverse effects of elevated CO_2_ on calcification, while *Stylophora pistillata* had reduced rates under laboratory conditions^25^.

In this study, we investigated the impacts of ocean acidification on zooplankton capture rates in a coral species known for its voracity in feeding, *Galaxea fascicularis*. This coral feeds on zooplankton by extending mesenterial filaments through the polyp mouth, capturing particles, and then either ingesting them or digesting them externally, outside the coelenteron^26^,^27^. Our study was based on four complementary field and laboratory experiments. They were conducted during two expeditions to fringing reefs in Papua New Guinea where CO_2_ seeps create natural pH gradients. We compared the morphology, behavior and feeding rates of *G. fascicularis* colonies grown in seawater with elevated CO_2_ (pH_T_ [total scale]~7.8, *p*CO_2_~760 μatm) against those grown at control CO_2_ (pH_T_~8.1, *p*CO_2_~420 μatm). We tested the following hypotheses: colonies acclimatized to elevated CO_2_ (1) have smaller polyps due to energetic constraints for calcification, (2) expand their polyps further, and (3) have increased rates of heterotrophy. We also tested for (4) food selectivity in *G. fascicularis* as a function of CO_2_ levels, and (5) whether the neurotransmitter receptor GABA_A_ was involved in the observed changes in the feeding ability of *G. fascicularis* under ocean acidification.

GABA (gamma-amino butyric acid) is one of many neurotransmitters within the central nervous system of cnidarians that helps regulate circadian rhythms in corals^28^,^29^ and modulates feeding responses in the cnidarian *Hydra vulgaris*^30^,^31^,^32^. Two receptors are associated with the neurotransmitter GABA: GABA_A_ and GABA_B_. There are multiple binding sites on each receptor with various possible agonists (chemicals that activate a biological response) and antagonists (chemicals that block the action of any agonist), which can attach to the binding site. The functioning of the GABA receptor GABA_A_ is of particular interest within ocean acidification research and has been linked to interference of neurotransmitter functioning in fish, mollusks, and other marine organisms^33^,^34^. Sensory and behavioral impairment of these organisms can effectively be reversed with one of the antagonist to the GABA_A_ receptor, gabazine, although it has never been tested in corals. *G. fascicularis* colonies under ocean acidification were treated with gabazine to determine its possible influences on heterotrophy.

*G. fascicularis* fragments used in this study have been exposed to high CO_2_ conditions their entire life; therefore, all observations of feeding behavior of *G. fascicularis* reflect heterotrophy of corals with life-long acclimation to ocean acidification.

## Results

### Feeding rates

**Figure 1:**
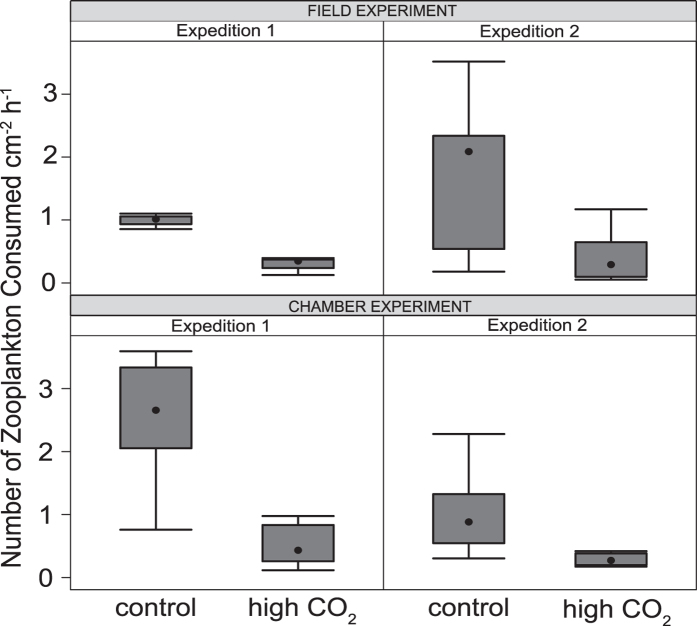
Rates of heterotrophy in the coral *Galaxea fascicularis* in all experiments from two methods (field and chamber), two expeditions, and two CO_2_ levels (control and high CO_2_).

*G. fascicularis* colonies acclimatized to high CO_2_ conditions (average pH_T_ of 7.8) consumed less zooplankton compared to colonies under control conditions (pH_T_ 8.1; Fig. 1). This result was consistent for all experiments across methods and expeditions. The difference in the total number of zooplankton consumed per surface area was statistically different between CO_2_ levels, but not between methods (i.e. field versus chamber), or between expeditions (Table 1). The interaction between method and expedition had a significant influence on the total number of zooplankton consumed, although there was no difference for the main effect variables of method and expedition (Table 1).

Following the observation of reduced feeding rates during the first expedition, in the second expedition we assessed whether the reduced heterotrophy was caused by CO_2_-induced impairment of neurotransmitters. The addition of gabazine during the chamber experiment from expedition 2 had no significant impact on the feeding rates (one-way ANOVA: F_(2,22)_ = 0.51; P = 0.48). Thus, heterotrophy rates under high CO_2_ were not restored by the treatment with gabazine, the GABA_A_ receptor antagonist.

### Composition of consumed food and selective feeding

**Figure 2:**
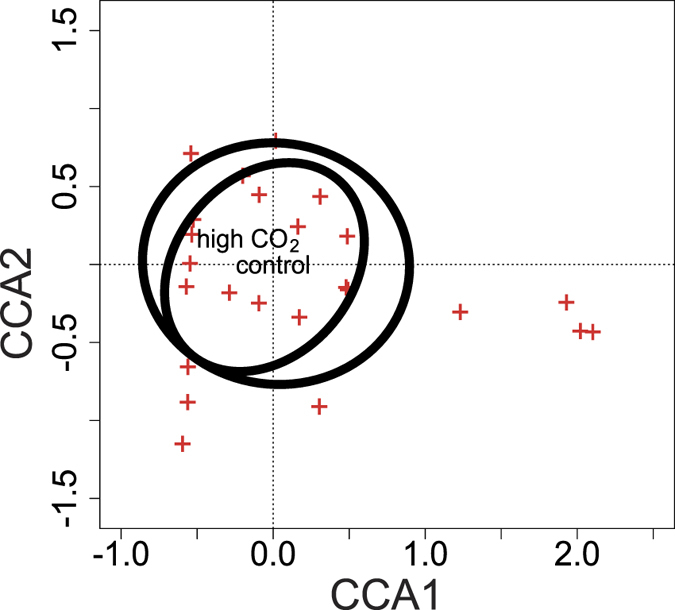
Community analysis of zooplankton consumed under contrasting CO_2_ regimes. Ordination plot from a canonical correlation analysis (CCA).

Although the total number of zooplankton consumed was different between CO_2_ levels, the types of zooplankton consumed by *G. fascicularis* were not different between CO_2_ levels. Taxonomic richness of the zooplankton prey consumed was not different between CO_2_ levels (three-way ANOVA: F_(1,13)_ = 2.74; P = 0.10), although it was higher in the chamber experiments compared to the field experiments (F_(1,15)_ = 20.2; P < 0.001), and higher in expedition 2 compared to expedition 1 (F_(1,15)_ = 8.17; P = 0.006). Multivariate community analyses on the prey consumed by corals supported these results and indicated that the zooplankton community consumed was also not different between CO_2_ levels (three-way ANOVA: F_(1,56)_ = 1.45; P = 0.14; Fig. 2; supplementary information Figure S2), although differed between methods (F_(1,56)_ = 2.86; P = 0.003), expeditions (F_(1,56)_ = 14.5; P = 0.001), and the interaction across the two variables (F_(1,56)_ = 2.95; P = 0.005).

**Figure 3:**
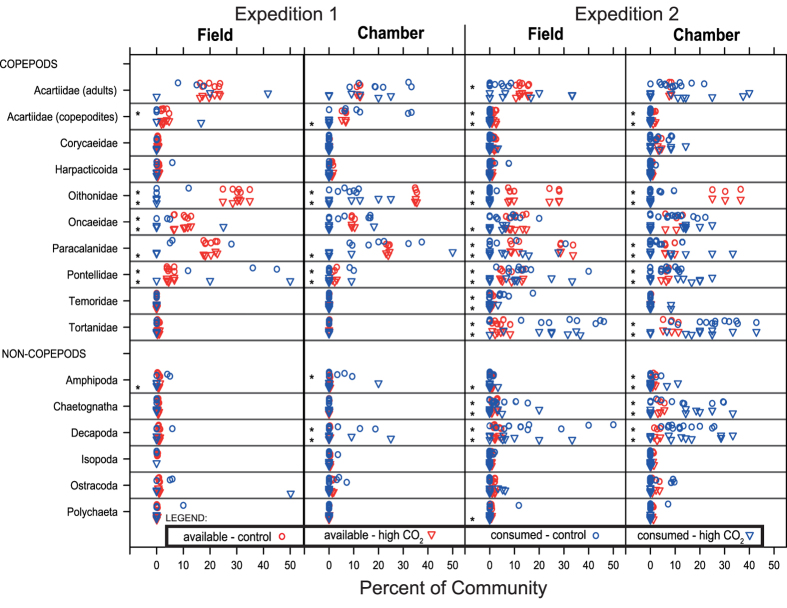
The percent composition of the top available and consumed zooplankton taxa is shown for both expeditions, methods, and between CO_2_ levels. Plots for the 16 most commonly consumed zooplankton taxa compare the percent of each taxon consumed by the coral represented in the coelonteron (blue symbols) to the percent of the community that each zooplankton is available in the water column (red symbols). Each zooplankton taxon has two rows, with the top row (circles) representing the control site and the bottom row (triangles) representing the elevated CO_2_ site. Each panel represents a separate experiment (two expeditions and two methods). Asterisks indicate a significant difference between the percent consumed and percent available in the water column (t-tests, p-value < 0.05).

The types of prey identified in the coelenteron of dissected corals had much lower taxonomic richness than the plankton available in the water column: corals contained only 11–17 zooplankton taxa of the 26–33 taxa present in the water. Corals preferentially ingested some zooplankton taxa, including *Pontellidae* and *Paracalanidae* copepods, decapods, amphipods, and chaetognaths, whereas *Oithonidae* copepods that are abundant in the water column were scarce in the food consumed (Fig. 3).

Results from logistic regressions that examined the effects of elevated CO_2_, expedition, and method, on the probability that each zooplankton taxon may be consumed indicated slight variation in the rates of consumption of the various taxa in response to these factors (Table 2). There was no difference in selectivity between high CO_2_ and control corals for the most available and most frequently consumed zooplankton taxa. However, the rare *Acartidae* copepodites, *Harpacticoida*, *Isopoda*, *Ostracoda*, and *Polychaeta* appeared preferentially consumed at the control CO_2_ level. These taxa all represent a small proportion of the plankton available and consumed (<2%). Furthermore, consumption rates of several zooplankton taxa differed between expeditions and methods. For example, *Tortanidae* copepods were rarely consumed during the first expedition, and yet during the second expedition they constituted on average 30.2% of the coral diet in the field experiment and 22.6% in the chamber experiment. Similarly, uptake rates of decapods and chaetognaths were relatively high during the second expedition.

### Corallite size and polyp expansion between CO_2_ levels

No difference was observed in the size of *G. fascicularis* corallites between colonies originating at the seep and control sites (1-way ANOVA: F_(1,62)_ = 2.7, P = 0.11). Elevated CO_2_ also had no effect on polyp expansion of *G. fascicularis* at the seep and control sites, neither in the field nor in the chamber experiments. While coral polyps were not expanded more under elevated CO_2_ compared to control CO_2_ levels (4-way ANOVA: F_(1,124)_ = 1.1; P = 0.29), they were expanded significantly more in the field compared to the chamber experiments (F_(1,126)_ = 22.0; P < 0.001), and in expedition 2 compared to expedition 1 (F_(1,125)_ = 12.2; P < 0.001; see supplementary Table S1). Furthermore, corals expanded their polyps more at the end of each experiment compared to the beginning (F_(1,123)_ = 6.3; P = 0.013).

## Discussion

The observed effects of ocean acidification on heterotrophy in the stony coral *Galaxea fascicularis* contradicted our initial hypothesis. We expected corals to ingest more zooplankton under high CO_2_. Instead, we found that food consumption rates were reduced under elevated CO_2_, both in the field and in chamber experiments, and during two expeditions. Since the colonies in our high and ambient CO_2_ treatments had been subjected to life-long exposure to their respective CO_2_ environments, this study presents the first investigation of heterotrophy in corals that were fully acclimatized to elevated CO_2_ throughout their entire post-settlement lives.

The taxonomic composition of the zooplankton consumed by *G. fascicularis* was different compared to the zooplankton community available to the corals. Such selectivity is known for corals^12^. Selectivity may be indicative of plankton behavior; for example, some zooplankton taxa swim more slowly or clumsily making it easier to capture them, while some taxa have chemical defenses that make them unpalatable to corals^35^. Whether it is from their own choosing or more from the behavior or chemical defenses of the zooplankton, there was strong selection for certain zooplankton, and this selectivity appeared to be largely unaffected by CO_2_ treatments, with the exception of only a few uncommon taxa (Table 2).

Selectivity results may be slightly biased towards larger zooplankton taxa since smaller groups digest faster than larger zooplankton^36^. However, the feeding time in this study was purposely chosen to be one hour so that complete digestion could be avoided. Complete digestion takes hours to days, and even small nauplii are still recognizable after only 60 minutes in the coelenteron^12^,^26^,^36^,^37^. Furthermore, since the mesh size of the plankton net was 100 μm, the smallest zooplankton types were excluded from the experiment. In fact, most zooplankton consumed were easily identifiable to species level even when partially digested, hence the category ‘unidentified consumed zooplankton’ represented only 13% of the items retrieved from the coelonteron.

*G. fascicularis* consumed less zooplankton in the high CO_2_ water despite having the same access to food, the same state of polyp expansion, and the same corallite sizes between CO_2_ treatments. The reasons for the observed reduction in feeding rates could be many, however our study negated several potential causes. Reduced heterotrophy was not caused by a reduction in corallite size since *G. fascicularis* corallites were the same size between CO_2_ levels, even though exposure to elevated CO_2_ reduces corallite sizes in some other coral species^38^. For example, the temperate coral *Oculina patagonica* showed smaller corallites at elevated CO_2_ due to high energetic costs for calcification; however, after one month of acidic conditions the skeleton completely dissolved and polyp sizes increased when calcification ceased and the resulting free energy was channeled into somatic growth^39^. With respect to *G. fascicularis,* it is possible that net calcification rates may change under ocean acidification conditions despite the morphology of the corallites remaining similar for both CO_2_ levels.

Reduced heterotrophy was also not caused by a difference in polyp expansion, which remained unaffected by ocean acidification for *G. fascicularis.* In contrast, another study observed that polyps from the coral *P. lutea* extended further under high CO_2_ conditions^24^. During the second expedition, however, *G. fascicularis* polyps were expanded more, which happened to occur during a new moon compared to the first expedition that had a full moon. Corals are known to feed differently with the lunar cycle, coinciding with lunar effects on zooplankton migration patterns^28^,^40^. Also, polyps were expanded more in the field experiments compared to the chamber experiment, probably because the corals were undisturbed in the field.

A deficiency in the functioning of GABA_A_ neurotransmitter receptors in *G. fascicularis* was also not a likely cause for the observed reduction in heterotrophy. Gabazine plays a role in *Hydra vulgaris* feeding response^31^, therefore we expected it to also influence coral feeding behavior of *G. fascicularis* since both of these cnidarians share similar nervous systems. Despite our predictions, the treatment of *G. fascicularis* with gabazine yielded no change in coral heterotrophy. The effect of ocean acidification on coral neurotransmitters cannot be completely excluded, however, because different chemicals besides gabazine may bind to the neurotransmitter receptors (e.g. the agonist muscimol and the antagonist bicuculline)^32^. To thoroughly understand the effect of ocean acidification on neurotransmitters of *G. fascicularis*, the reactions of other receptor antagonists and agonists to elevated CO_2_ need to be evaluated.

Additional experiments are needed to reveal the underlying mechanisms responsible for the reduced feeding rates in *G. fascicularis*. Potential causes or contributors that deserve further study include reduced particle retention, changes in cellular homeostasis of the tentacle cells, reduced nematocyst functioning, altered mucus production, physiological stress that makes them less capable to feed, an increase in autotrophy, and potential changes in plankton behavior, as briefly outlined here. *G. fascicularis* exerted similar effort to capture zooplankton between CO_2_ levels by extending their polyps to the same level. That they ingested fewer food particles in ocean acidification conditions may reflect upon the polyps’ ability to capture food. Food retention may be reduced if the functionality of their stinging cells (nematocysts) is disrupted^41^,^42^. Nematocyst performance may be vulnerable to changes in pH since the acid-base balance in cells corresponds to the intracellular concentration of free H^+^ ions. A study on the jellyfish *Pelagica noctiluca* indicated that the cell homeostasis of nematocysts is profoundly compromised by acidification of the surrounding seawater impairing the cells’ discharge capability^43^. Although cellular homeostasis in nematocysts may vary between jellyfish and corals, nematocyst functioning may be impaired for corals under ocean acidification and merits further investigation.

Another possible cause for the observed reduced feeding rates could be that the polyps themselves lose their ability to retain food particles. Food particles may be stung or killed, but the mucosal or tentacular action of the polyps may not trap the particles, resulting in the loss of prey items^26^. Mucus enhances coral heterotrophy^44^, therefore heterotrophy will likely be vulnerable to any changes in mucus production, but nothing is known about how ocean acidification may affect coral mucus.

It is perceivable that *G. fascicularis* may also have reduced rates of heterotrophy in response to a reduced energy demand. Elevated CO_2_ enhances the photosynthetic-derived energy supply in some coral species, and this energy is available to support critical functions like calcification. Coral calcification is generally considered to decline with elevated CO_2_ levels^45^, although some studies report parabolic and even positive calcification responses to ocean acidification conditions^46^,^47^. However, corals are more nutrient limited than carbon limited in oligotrophic and shallow (high-light) environments^2^. Furthermore, feeding rates of corals only reach saturation when food concentrations are high, with heterotrophy generally more efficient in oligotrophic habitats^48^. Considering that *G. fascicularis* from the CO_2_ seep sites live in a nutrient-poor and high-light environment, it is highly unlikely that feeding becomes saturated and their need for essential nutrients not attained from photosynthesis would still be prevalent. Therefore, *G. fascicularis* would likely continue to feed on zooplankton at the CO_2_ seep sites if they were still capable even under an increased carbon supply from photosynthesis.

Regardless of the underlying mechanisms, reduced heterotrophy under elevated CO_2_ will have biological impacts on corals. Growth, reproduction, zooxanthellae maintenance^49^, and other metabolic processes depend on nitrogen, phosphorus, and other essential trace elements, which are exclusively attained through heterotrophy^6^,^50^,^51^,^52^. We are only starting to understand the long-term impacts of ocean acidification on tissue growth, phototrophy, respiration, heterotrophy, and their energetic interdependencies, in selected species of coral. Many but not all coral species increase their rates of photosynthesis at higher *p*CO_2_ levels^53^. Reduced heterotrophy may also impact coral lipid content and fatty acid composition, since they are co-determined by zooplankton consumption^54^. Furthermore, lower feeding rates may slow skeletal and tissue growth considering that growth is positively correlated with rates of heterotrophy for several coral species^6^,^52^, so lower feeding rates may slow growth. Heterotrophy is certainly beneficial to corals and yet clearly heterotrophy declines for *G. fascicularis* under elevated CO_2_. Any potential impact to their basic biology warrants further research.

Despite the remaining knowledge gaps, decreased heterotrophy will have important implications for the health and resilience of corals. As ocean conditions increasingly become unfavorable for many coral species, their ability to react to such stress will become imperative to their survival. Some coral species will persist while others will not, and our data show that some *G. fascicularis* colonies are able to survive under high CO_2_ in the field, despite their lifetime exposure to elevated CO_2_ conditions and associated reduced zooplankton feeding rates. However, it was beyond the scope of this study to measure their physiology (tissue biomass, lipid content, calcification rates, or other biophysical parameters indicative of their overall health). Such measurements should be conducted to better understand coral long-term survivability under ocean acidification.

## Methods

### Study site

The feeding experiments were conducted at Upa-Upasina Reef, a fringing reef in Milne Bay Province, Papua New Guinea, where a natural volcanic CO_2_ seep provides gradients in seawater pH^55^. A spatial map of the seawater carbonate chemistry, along with a detailed description of the Upa-Upasina high CO_2_ and control site can be found in Fabricius *et al.*^55^,^56^. *G. fascicularis* colonies were collected near the seep site where seawater approximates 7.8 pH_T_ (total scale), and from a control site with control CO_2_ at ~8.1 pH_T_. The chamber feeding experiments were conducted aboard the back deck of the ship while moored near Upa-Upasina Reef, with *G. fascicularis* fragments that were freshly collected from the reef. The field and chamber experiments were conducted during two ship expeditions to the site (12–14 April 2014 and 18–20 November 2014).

### Seawater carbonate chemistry

The carbonate chemistry for the field sites varied through time and long-term measurements have been reported in previous literature^56^. Additionally, seawater pH at total scale (pH_T_) was recorded at the control and elevated CO_2_ sites for several days surrounding the commencement of the feeding experiments using SeaFET pH sensors (Supplementary Information Figure S3). pH_T_ values had similar ranges compared to previous expeditions^55^,^56^. Water samples were also collected, fixed with saturated mercuric chloride solution (HgCl_2_), and later analyzed for their dissolved inorganic carbon (DIC: μmol kg^−1^) and total alkalinity (*A*_T_: μmol kg^−1^) using the Versatile Instrument for the Determination of Total Inorganic Carbon and Titration Alkalinity (VINDTA 3C).

Carbonate chemistry was also measured for the seawater used for the chamber experiments and water temperature (°C) was recorded on site. Water samples saturated with HgCl_2_ were stored and later measured for DIC and *A*_T_. The water temperature was 25 °C at the time the samples were analyzed in the laboratory for its carbonate chemistry using the VINDTA 3C. DIC and *A*_T_ were used to calculate other seawater parameters (Table 3), including pH at total scale (pH_T_), partial pressure of carbon dioxide (*p*CO_2_: μatm), bicarbonate (HCO_3_^−^: μmol kg^−1^), carbonate (CO_3_^2−^: μmol kg^−1^), aqueous carbon dioxide (CO_2(*aq*)_: μmol kg^−1^), the saturation state of calcite (Ω_CA_), and the saturation state of aragonite (Ω_AR_), using the Excel macro CO2SYS^57^ under the constraints set by Dickson and Millero (1987)^58^.

### Food collection

Zooplankton were freshly collected via plankton net tows from the control site at approximately 9 pm, i.e. 2–3 h after sunset, and shortly before the start of the field and the chamber experiments. Each net tow was very slow to minimize stress to the zooplankton. Live samples were handled with care and only living zooplankton were used as food for corals (i.e. zooplankton still suspended in the water column and actively swimming. No zooplankton that had settled at the bottom of the collection container were used). Three to six zooplankton samples were preserved in 4% formalin and kept as references to determine variation in the number and taxonomic composition of zooplankton between samples.

### Field feeding experiment

Tents of 100 μm plankton mesh and 25 cm base diameter (approximately 8 L volume) were used to contain zooplankton close to corals for the duration of the feeding experiment. Five tents were placed over separate *G. fascicularis* colonies each at the high CO_2_ and the control sites. To prevent corals from consuming zooplankton that are naturally in the water column, the tents were deployed during daylight when zooplankton numbers are low and demersal zooplankton have not emerged into the water column yet. At approximately 9 pm, SCUBA divers injected three 60 ml syringes of freshly collected and concentrated zooplankton into each tent. Polyp expansion (25, 50, 75 or 100 percent expanded) was recorded at the beginning and end of the feeding period. After approximately one hour, the tents were removed and a fragment of each colony was extracted with a hammer and chisel and preserved in 4% formalin. The field experiments were conducted once during expedition 1 (three replicate coral colonies per CO_2_ level), and twice on two consecutive nights during expedition 2 (five replicate coral colonies per CO_2_ level for both nights). The plankton fed to the corals during the second expedition had similar composition and concentration in the two consecutive nights, so the results from both nights were pooled together and considered one experiment.

### Chamber feeding experiment

*G. fascicularis* fragments were collected from both the high CO_2_ site and the control site. They were placed in flow-through aquaria for four days to recover. The aquaria consisted of two 60 L bins with an outboard pump supplying a constant inflow of fresh seawater. For 12 hours prior to the feeding experiment, 100 μm mesh was placed over the input valve to starve *G. fascicularis*, allowing any previously consumed food to be digested. Three hours prior to the feeding experiment, each coral fragment was transferred onto a raised grid platform in individual cylindrical incubation chambers (89 mm diameter, 106 mm height, 637 ml volume) without exposing them to air. Corals collected from the seeps were placed in the chambers filled with seawater from the seep site, while those from the control site were placed in chambers filled with seawater from the control site (seawater carbonate chemistry for chamber experiments found in Table 3).

Chambers were 80% immersed in a water bath. Airspace in the chamber and a hole in its upper lid facilitated gas exchange. To generate a current within the chamber, a battery driven pulley system activated magnetic stirrer bars underneath the grid^53^. *G. fascicularis* were fed at around 9 pm. Taking care to supply only living zooplankton, concentrated zooplankton was injected through a hole in the top lid of the chamber with a volumetric pipette. The zooplankton concentration was lower during the second expedition compared to the first, so a larger volume of plankton solution was inserted into the chamber during the second (30 ml) compared to the first expedition (20 ml). An additional three samples of the food were preserved in 4% formalin and kept as references. The feeding experiment was conducted in the dark, although red light was used for a few minutes at the commencement and cessation of the experiment to assess their state of polyp expansion. *G. fascicularis* fed for approximately one hour and then each coral piece was removed and immediately stored in 4% formalin. The chamber experiment was conducted once through an initial pilot study during expedition 1 (7 replicate coral colonies per CO_2_ level), and repeated during expedition 2 with additional replicates (12 replicate coral colonies per CO_2_ level).

To determine if elevated CO_2_ interferes with neurotransmitter receptor functioning, six of the coral fragments per CO_2_ treatment were exposed to gabazine (SR-95531, Sigma-Aldrich) at a concentration of 4 mg L^−1^ seawater for 30 min (chamber experiment, second expedition). Coral fragments were gently washed and transferred into their chambers filled with gabazine-free seawater. The other six colonies per CO_2_ treatment were exposed to the same handling procedure, but their 30 min transfer was into a container without gabazine. Experiments were then conducted as outlined above.

### Food samples for corals

Food samples given to corals were compared within and between experiments. Food samples given to each replicate coral fragment were similar in quantity and composition within each experiment, and they were not different between high CO_2_ (7.8 pH_T_) and control treatments (8.1 pH_T_) and replicates. However, food samples varied in quantity and composition between the four field and chamber experiments. Details about the analysis of food samples are in the supplementary information, including Figure S1.

### Laboratory analysis

Coral consumption was measured through coelenteron content analysis. *G. fascicularis* fragments were removed from formalin and placed in freshwater. Every polyp coelenteron was probed using a tungsten needle and dissecting forceps. Extracted zooplankton were identified to their major taxonomic groups. Total corallite number and corallites containing food particles were enumerated. Each coral fragment was photographed and the surface area calculated within the image-processing program, ImageJ. Corallite size was calculated by dividing the surface area of each coral fragment by the number of corallites.

### Statistics

All statistical analyses were computed in R, version 3.2.2 (R Development Core Team, 2015). Generalized linear models (GLMs) were used to determine if: (1) the number of zooplankton consumed (standardized by surface area) differed across CO_2_ regimes (seep vs. control), expedition (one vs. two), or methods (field vs. chamber), (2) species richness (Shannon-diversity index) of the zooplankton taxa consumed by corals differed between CO_2_ regimes, expedition, or methods, (3) gabazine affected coral feeding rates, (4) zooplankton concentration in the food samples was different between each of the experimental runs, (5) corallite sizes were different between corals originating from seep and control sites, and (6) polyp expansion differed across CO_2_ levels, methods, expeditions, or from the beginning to the end of the experiment. Appropriate data distributions and link functions were chosen for each GLM. Model assumptions of independence, homogeneity of variance, and normality of error were evaluated through diagnostic tests of leverage, Cook’s distance, and dfbetas^59^. Checks for all GLMs indicated that no influential data points or outliers existed in the data and model assumptions were met. ANOVAS (Type II) were used to determine the minimal adequate GLM with the ‘Anova’ function in the R library ‘car’ (version 2.1-1)^60^. The effects of the explanatory variables on the response variables were then reported based on these GLMs.

Canonical correspondence analysis (CCA) was used to determine if the zooplankton community composition of the food available to the corals, and the food consumed by the corals, differed in relation to the explanatory variables (CO_2_, expedition, method). To account for many zeros in the data where some zooplankton taxonomic groups were rarely present or rarely consumed, the community data was standardized using the Hellinger (square root) method within the decostand function of the vegan package in R^61^. A Monte-Carlo permutation test was used to determine the optimal CCA model and to assess the significance of the variation in species composition attributable to the explanatory variables (CO_2_, expedition, method).

For each zooplankton taxon, its percent representation in the coral coelenteron content was compared against its percent in the available food using two-tailed t-tests, assuming unequal variances between samples. Logistic regressions were used to model the response of each zooplankton taxon contained in the corals to the explanatory variables of CO_2_, expedition, and method. Logistic regressions use binary data of ‘successes’ and ‘failures’. In this example, ‘success’ equals the probability of each taxon being consumed (p), and ‘failure’ equals the probability of not being consumed (1-p). Logistic regressions are within the framework of GLMs and use log-odd-ratios, defined by the logit link function, to estimate the (log) odds of each taxon being consumed under each independent variable. GLMs with a binary data distribution and logit link function were checked for overdispersion. Overdispersion (residual deviance greater than the residual degrees of freedom) existed, so the data distribution was changed to quasibinomial. Anovas with a Chi-square test were applied to the results of each GLM for each zooplankton taxon.

## Additional Information

**How to cite this article**: Smith, J. N. *et al.* Reduced heterotrophy in the stony coral *Galaxea fascicularis* after life-long exposure to elevated carbon dioxide. *Sci. Rep.* **6**, 27019; doi: 10.1038/srep27019 (2016).

## Supplementary Material

Supplementary Information

## Figures and Tables

**Table 1:** Results of a generalized linear model regression of coral feeding rates in response to method, expedition, CO_2_, and their interaction terms.

Factors and Interactions	F_(df,df)_	P-value
Method	F_(1,61)_ = 0.46	0.50
Expedition	F_(1,60)_ = 1.9	0.18
CO_2_	F_(1,62)_ = 51.9	<0.001*
Method: Expedition	F_(1,57)_ = 9.4	0.003*
Method: CO_2_	F_(1,59)_ = 0.39	0.53
Expedition: CO_2_	F_(1,58)_ = 0.25	0.62
Method: Expedition: CO_2_	F_(1,56)_ = 0.48	0.49

**Table 2:** Probability for each of the 16 most common zooplankton taxon to be consumed by *Galaxea fascicularis,* as a function of CO_2_ (seep vs. control), expedition (one vs. two), method (field vs. chamber), and the interactions of these parameters (three-way interactions were non-significant for all taxa and are not shown).

Taxon	CO_2_		Expedition		Method		CO_2_: Expedition		CO_2_: Method		Expedition: Method	
	Χ^2^	p-value	Χ^2^	p-value	Χ^2^	p-value	Χ^2^	p-value	Χ^2^	p-value	Χ^2^	p-value
**COPEPODS**												
Acartiidae (adults)	8.78	0.679	8.06	**0.011**	7.87	0.196	7.52	0.074	7.29	0.15	6.92	0.071
Acartiidae (copepodites)	5.28	**<0.001**	2.44	**<0.001**	2.26	0.003	2.26	0.999	1.26	**<0.001**	1.26	0.999
Corycaeidae	2.71	0.889	2.15	**<0.001**	1.58	**<0.001**	1.58	1.000	1.57	0.611	1.57	0.999
Harpacticoida	0.81	**<0.001**	0.81	0.907	0.63	**0.001**	0.63	0.999	0.63	0.999	0.60	0.169
Oithonidae	5.01	0.464	2.98	**<0.001**	2.54	**<0.001**	2.27	**0.008**	2.11	**0.034**	2.08	0.441
Oncaeidae	6.33	0.151	6.28	0.464	6	0.077	6.00	0.932	6.00	0.943	5.90	0.303
Paracalanidae	10.1	0.585	9.46	**0.047**	9.29	0.324	8.27	0.142	8.23	0.654	7.93	0.179
Pontellidae	13.9	0.614	13.6	0.199	12.6	**0.029**	12.4	0.412	11.9	0.110	11.6	0.217
Temoridae	2.62	0.171	2.17	**<0.001**	2	**0.025**	2.00	0.999	1.47	**<0.001**	1.47	0.999
Tortanidae	18.4	0.989	8.70	**<0.001**	8.37	0.096	8.38	1.000	8.03	0.083	8.03	1.000
**NON-COPEPODS**												
Amphipoda	2.98	0.922	2.70	**0.030**	2.61	0.218	2.56	0.385	2.45	0.178	2.45	0.885
Chaetognatha	8.83	0.656	6.23	**<0.001**	4.94	**<0.001**	4.94	0.999	4.94	0.873	4.94	0.999
Decapoda	9.24	0.173	7.72	**<0.001**	7.7	0.695	7.69	0.771	7.46	0.177	7.25	0.196
Isopoda	0.35	**<0.001**	0.34	0.203	0.34	0.390	0.34	0.999	0.34	0.999	0.26	0.545
Ostracoda	2.23	**0.002**	2.22	0.574	2.22	0.766	2.06	**0.013**	1.74	**<0.001**	1.45	**<0.001**
Polychaeta	1.41	**<0.001**	1.41	0.841	1.25	**0.042**	1.25	0.999	1.25	0.999	1.14	0.082

Χ^2^ (with df = 1 for all parameters) and p-values from the logistic regression analysis are presented, with bold print indicating significances at p < 0.05.

**Table 3:** Seawater carbonate chemistry of the chamber experiments with dissolved inorganic carbon (DIC) and total alkalinity (*A*_T_) measured from water samples fixed with saturated mercuric chloride solution (HgCl_2_).

Expedition	Treatment	pH_T_	Temperature (°C)	*A*_T_ (μmol kg^−1^)	DIC (μmol kg^−1^)	*p*CO_2_ (μatm)	HCO_3_^−^ (μmol kg^−1^)	CO_3_^2−^ (μmol kg^−1^)	CO_2(*>aq*)_ (μmol kg^−1^)	Ω_CA_	Ω_AR_
1	control	8.05	28.0	2206	1887	381	1652	225	9.7	5.54	3.69
1	elevated-CO_2_	7.70	28.0	2282	2135	1028	1987	121	26.3	2.97	1.98
2	control	8.08	29.5	2270	1938	359	1693	236	9.5	5.78	3.84
2	elevated-CO_2_	7.75	29.5	2336	2171	906	2015	132	24.0	3.25	2.15

DIC and *A*_T_ were inputted into the Excel macro CO_2_SYS and used to calculate pH at total scale (pH_T_), partial pressure of carbon dioxide (*p*CO_2_), bicarbonate (HCO_3_^−^), carbonate (CO_3_^2−^), aqueous carbon dioxide (CO_2(*aq*)_), the saturation state of calcite (Ω_CA_), and the saturation state of aragonite (Ω_AR_).
