# Supplementary material for: Reduced heterotrophy in the stony coral Galaxea fascicularis after life-long exposure to elevated carbon dioxide
Source: Sci Rep. 2016 Jun 3;6:27019. doi: 10.1038/srep27019 (PMC4891704; doi:10.1038/srep27019)
Supplement: Supplementary Information [file srep27019-s1.pdf]

Supplementary Information

Manuscript Title: Reduced heterotrophy in the stony coral *Galaxea fascicularis* after life-long exposure to elevated carbon dioxide

Authors: Joy N. Smith, Julia Strahl, Sam H.C. Noonan, Gertraud M. Schmidt, Claudio Richter, Katharina E. Fabricius

Supplementary Figures

Supplementary Figure S1. Food samples given to corals.

a.) The food concentration of zooplankton in the water was greater in expedition 1 compared to expedition 2, and greater in the chamber experiments compared to the field experiments.

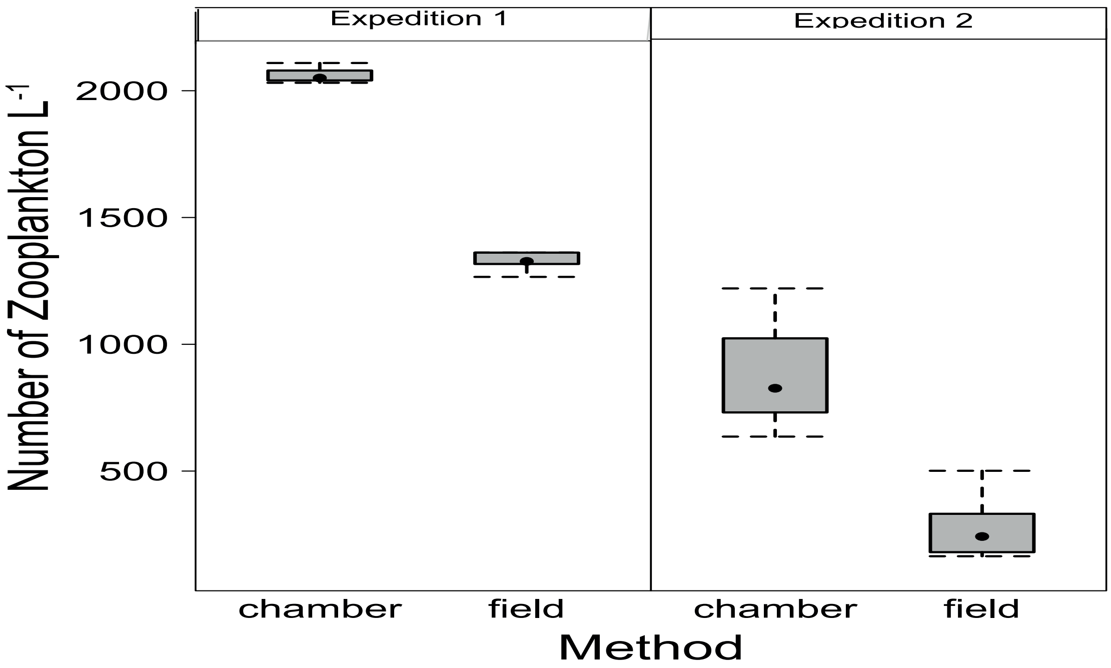

b.) Results from a canonical correspondence analysis (CCA) reveal that the composition of food samples given to corals differed between expedition 1 and expedition 2.

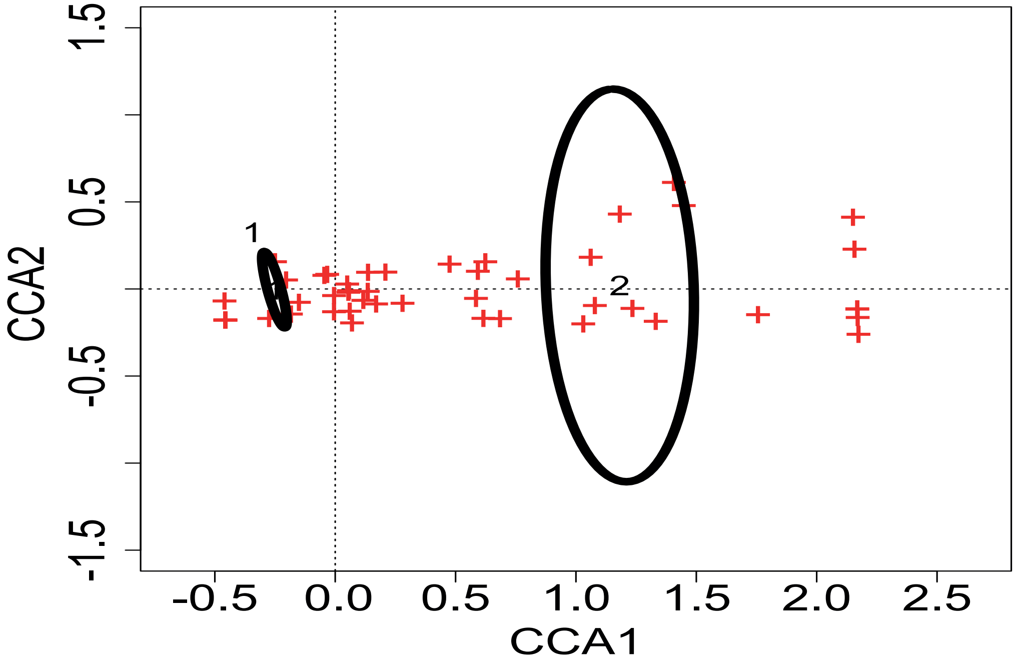

Supplementary Figure S2. Zooplankton community consumed by corals vary between expedition and method

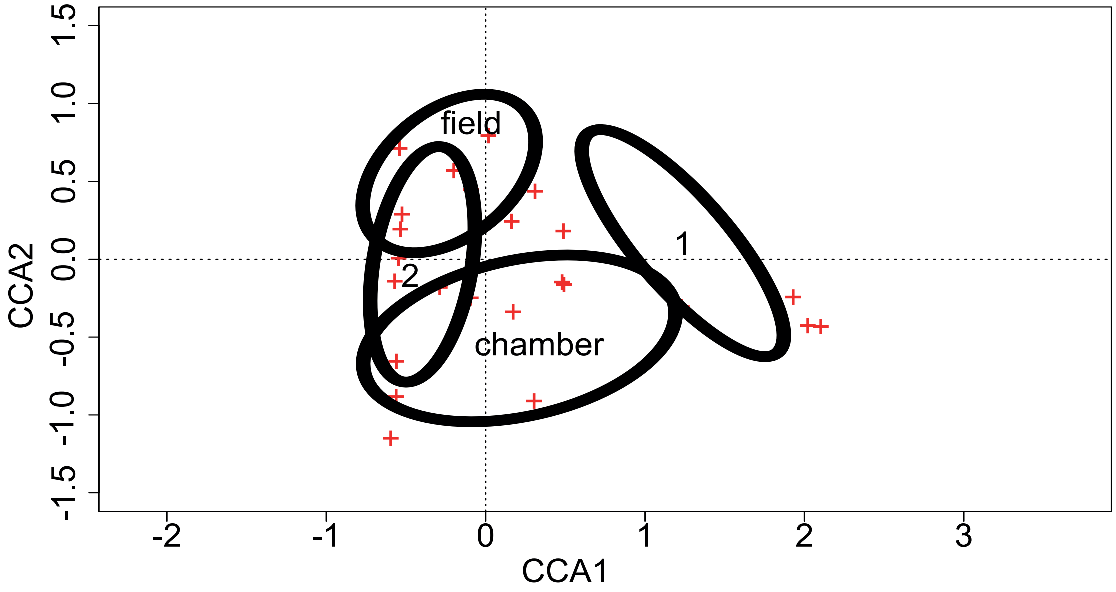

Supplementary Figure S3. Time series of seawater pH_T for the field site during both expeditions.

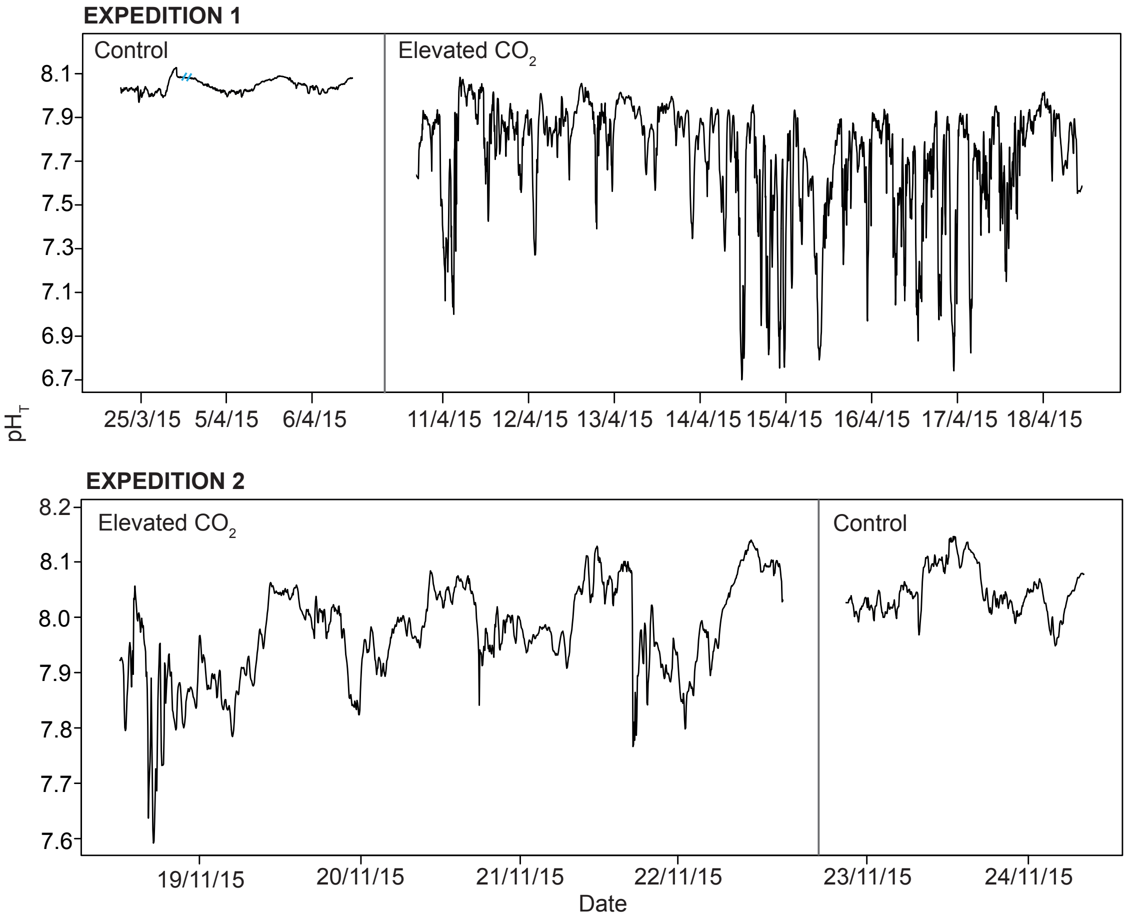

Supplementary Tables

Table 1S. Results of GLM regression for polyp expansion

Factors and Interactions	$F_{(df,df)}$	P-value
Method	$F_{(1,126)} = 22.0$	< 0.001 *
Expedition	$F_{(1,125)} = 12.2$	< 0.001 *
CO_2	$F_{(1,124)} = 1.2$	0.269
Time Point	$F_{(1,123)} = 6.3$	0.013 *
Method: Expedition	$F_{(1,122)} = 2.4$	0.124
Method: CO_2	$F_{(1,121)} = 2.1$	0.147
Expedition: CO_2	$F_{(1,120)} = 3.8$	0.054
Method: Time Point	$F_{(1,119)} = 0.003$	0.952
Expedition: Time Point	$F_{(1,118)} = 1.0$	0.329
CO_2: Time Point	$F_{(1,117)} = 0.4$	0.531
Method: Expedition: CO_2	$F_{(1,116)} = 0.7$	0.394
Method: Expedition: Time Point	$F_{(1,115)} = 0.2$	0.673
Method: CO_2: Time Point	$F_{(1,114)} = 1.8$	0.183
Expedition: CO_2: Time Point	$F_{(1,113)} = 0.1$	0.711
Method: Expedition: CO_2: Time Point	$F_{(1,112)} = 0.001$	1.000

Supplementary Text

Food samples for corals

To determine the variance between food samples between replicates, treatments, field and chamber experiments, and the two expeditions, the coefficient of variation (CV) was calculated for each zooplankton taxonomic group, as well as for the total number of zooplankton. Food samples given to corals were similar in quantity and composition within each experiment. When comparing food samples across replicates within the same experiment, coefficient of variance (CV) values for the total number of zooplankton and for all dominant taxonomic groups were

always <1. In other words, the food samples had similar food concentrations in each replicate syringe for each experiment. Only rare taxonomic groups (<1% of the entire community) had high variation between replicate food samples, i.e. CV>1.

Generalized linear models were used to compare zooplankton quantity between experiments and canonical correspondence analyses were used to compare the composition of zooplankton in the food samples between experiments. Zooplankton quantity of the food samples was different between each experiment and the composition of the food samples differed between expeditions (Supplementary Fig S1). More specifically, food concentrations were significantly different between the chamber and field experiments (3-way ANOVA: $F_{(1,16)} = 102$; $P < 0.001$) and between the two expeditions ($F_{(1,15)} = 311$; $P < 0.001$). There was no difference in food concentrations between the two field experiments conducted on consecutive nights during the second expedition ($F_{(1,14)} = 1.9$; $P=0.19$); therefore, those experiments were grouped together for all further analysis. Food concentrations were higher for expedition 1 compared to expedition 2, and greater for the chamber experiments compared to the field experiments. The mean food concentrations (number of zooplankton L^{-1}±SE) for each experiments were: expedition 1 - chamber, 2063.5±23.5; expedition 1- field, 1342.7±26.3; expedition 2 – chamber, 894.3±172.1; and expedition 2-field, 276.8±52.4. Despite lower food concentrations in expedition 2, species richness was actually significantly higher in expedition 2 compared to expedition 1 (two-way ANOVA: $F_{(1,16)} = 9$, $P < 0.001$), with an average ± SE of available prey types in expedition 2 being 26±2.4 and 33±0.6 in expedition 1. Species richness of available food types was not different between

methods (two-way ANOVA: $F_{(1,15)} = 9$, $P = 0.06$). A community analysis of the food samples confirms that the zooplankton communities were significantly different between expeditions (two-way ANOVA applied to CCA results: $F_{(1,14)} = 12.1$; $P = 0.001$), but not methods ($F_{(1,14)} = 12.1$; $P = 0.62$). The quantity and composition of zooplankton available to *Galaxea fascicularis* varied between experiments, but they were similar within each experiment and across the CO_2 treatments, thus ocean acidification affects on coral feeding behavior can still be evaluated.

Community analysis of zooplankton consumed by corals for different expeditions and methods

Although the community consumed by *G. fascicularis* did not differ across CO_2 levels (Figure 2 from main text), it did differ depending on the expedition and method (chamber versus field experiments; Supplementary Figure 2).

Results from generalized linear models (GLM): effects of method, expedition, and CO_2 on polyp expansion

Polyp expansion of corals was different across methods, expedition, and from the beginning of the experiment to the end. However, polyp expansion did not differ across CO_2 regimes or any of the interaction terms (Supplementary Table S1).

pH of seawater for field experiments

Seawater pH at total scale (pH_T) was recorded for several days around the commencement of the feeding experiments. Measurements were collected at the

control and elevated CO_2 sites using SeaFET pH sensors and the data can be found in Supplementary Figure S3.
